# HLA-G Expression Is an Independent Predictor for Improved Survival in High Grade Ovarian Carcinomas

**DOI:** 10.1155/2014/274584

**Published:** 2014-05-27

**Authors:** M. J. Rutten, F. Dijk, C. D. Savci-Heijink, M. R. Buist, G. G. Kenter, M. J. van de Vijver, E. S. Jordanova

**Affiliations:** ^1^Academic Medical Center, Center of Gynaecologic Oncology, Amsterdam, The Netherlands; ^2^Department of Pathology, Academic Medical Center, Amsterdam, The Netherlands; ^3^Free University Medical Center, Center of Gynaecologic Oncology, Amsterdam, The Netherlands

## Abstract

Aberrant expression of human leukocyte antigens (HLA) class I has prognostic importance in various cancers. Here, we evaluated the prognostic value of classical (A/B/C) and nonclassical (G/E) HLA expression in 169 high grade epithelial ovarian cancer samples and linked that to clinicopathological characteristics and survival. Expression of HLA-A, -B/C, or -E was not correlated with survival. Survival was prolonged when tumours expressed HLA-G (*P* = 0.008) and HLA-G was an independent predictor for better survival (*P* = 0.011). In addition, HLA-G expression was associated with longer progression-free survival (*P* = 0.036) and response to chemotherapy (*P* = 0.014). Accordingly, high expression of HLA-G mRNA was associated with prolonged disease-free survival (*P* = 0.037) in 65 corresponding samples. Elevated serum-soluble HLA-G levels as measured by enzyme-linked immunosorbent assay in 50 matched patients were not correlated to HLA-G protein expression or gene expression nor with survival. During treatment, sHLA-G levels declined (*P* = 0.038). In conclusion, expression of HLA-G is an independent prognostic factor for improved survival in high grade epithelial ovarian cancer and a predictor for platinum sensitivity.

## 1. Introduction

Epithelial ovarian cancer (EOC) is the most lethal gynaecological cancer and the second cause of cancer related death among women [1]. Stage at diagnosis, result of debulking surgery, histological type, and response to chemotherapy all influence the prognosis [2–4]. All patients are treated with cytoreductive surgery and adjuvant combination chemotherapy, usually consisting of paclitaxel and carboplatin. Although response to treatment is high, approximately 70% of patients with advanced disease develop recurrence, suggesting that effectiveness of treatment protocols is low.

Morphologically and histologically, there are large differences within epithelial ovarian cancer, suggesting different patterns of development. Therefore, a classification based on a dualistic model of carcinogenesis is developed [5]. Type I tumours are low grade, are slowly growing, and develop from premalignant stage towards malignant lesion, while type II tumours are high grade, more aggressively growing, and often present at an advanced stage [6–8]. However, even within type II tumours, large differences exist regarding response to treatment and patient survival.

Molecular variations or interference of the immune system with the disease process may be accountable for this [9, 10]. As in other solid tumours like breast, colon, and cervical cancer, in epithelial ovarian cancer expression of human leukocyte antigens (HLA) is also associated with prognosis [9, 11–14]. The role of these molecules is to present intracellular peptides to cytotoxic T cells and herewith trigger an adequate immune response against the aberrant cells [15]. In contrast, nonclassical HLA expression is thought to play a role in immune tolerance by inhibiting natural killer (NK) cell-mediated lysis [16–21]. Studies have also indicated that soluble HLA-G (sHLA-G) could play a role in suppressing the functions of various immune competent cells [22–24].

Downregulation of classical HLA is associated with an unfavourable prognosis in ovarian cancer [25, 26]; however, the association of HLA-G with prognosis is controversial [12, 27–32]. Upregulation of HLA-G by IL6, IL8, or IL10 was suggested to help cancer cells evade the immune response by inhibition of NK cell- and CTL-mediated lysis [33–35]. However, the opposite was seen in melanoma cells [30]. The same controversies regarding prognosis are seen between studies analysing HLA-G expression in ovarian cancer. These controversies could be due to the fact that heterogeneous groups of patients were analysed [7, 36]. As HLA-G is frequently expressed in high grade ovarian tumours and almost never in low grade tumours, the role of this molecule could be different within these tumour types [37]. To optimize treatment regimens it is important to search for prognostic markers in homogenous cohorts of patients.

The purpose of this study was to evaluate whether expression of classical and nonclassical HLA influences survival in a cohort of 169 clinically well-characterized high grade epithelial ovarian cancer patients. In addition, we analysed HLA-G gene expression and serum sHLA-G concentration in all available matched frozen biopsies and serum samples, respectively.

## 2. Material and Methods

### 2.1. Patients and Material

This study includes patients treated for primary epithelial ovarian cancer in the Gynaecologic Oncologic Centre of the Academic Medical Centre Amsterdam and one of its referral hospitals, the Deventer Hospital, between 1993 and 2010. Haematoxylin/eosin (H&E) stained slides of the tumours were retrieved from the pathology department archives and were reviewed. Histological features, including histological grade, were assessed by an experienced pathologist (MV) blinded to the clinical data. In total, 169 patients with type II, high grade serous and undifferentiated tumours, were selected. Representative tumour areas were marked on the H&E slides to be cored for the array blocks. Furthermore, corresponding frozen tissue of 65 type II tumours was collected for RNA isolation. From 50 patients matched serum samples were available.

Clinical data of these patients were obtained from a prospectively maintained database at the Department of Gynaecologic Oncology at the Academic Medical Centre Amsterdam and missing data were abstracted from patient charts. Staging of the disease was done according to the criteria of International Federation of Gynaecologists and Obstetricians (FIGO). All patients were treated with primary debulking surgery (PDS) or interval debulking surgery (IDS) and a platinum based combination chemotherapy if indicated by disease stage. Patients who had more than one centimetre of residual tumour after primary debulking surgery underwent an interval debulking, solely when there was no progression during chemotherapy treatment. Progression-free survival (PFS) and disease specific survival (DSS) were calculated from the date of first surgery or start of chemotherapy to the date of progression, death, or last follow-up, respectively. Result of surgery was scored as no macroscopic disease or any residual tumour. Furthermore, sensitivity to platinum containing chemotherapy was classified according to moment of recurrence. When recurrence occurred within 6 months after the last cycle of chemotherapy was given, tumours were classified as platinum resistant. If recurrence occurred more than one year after the last cycle of chemotherapy was given, tumours were classified as platinum sensitive [38, 39]. The tissue and serum as well as the clinical data were used according to the guidelines of the Medical Ethical Committee of the Academic Medical Centre Amsterdam.

### 2.2. Tissue Microarray

To construct the tissue microarrays (TMA), formalin-fixed, paraffin-embedded (FFPE) tissue samples were collected from the pathology archive of both hospitals. We retrieved FFPE samples of primary tumour or metastasis at surgical intervention, which was either before treatment with chemotherapy or after 2 cycles of chemotherapy. Of each tumour, 3 representative 0.6 mm diameter cores were taken, resulting in 20 to 24 tumours on each TMA block (Beecher, Silverspring, MD, USA, TMA instrument). Sections of 4 *μ*m were obtained from each TMA block and placed on coated glass slides to allow for immunohistochemical staining.

### 2.3. Immunohistochemistry and Evaluation of Staining

For HLA class I staining, antibodies against HLA-A (HCA2) and HLA-B/C (HC10) heavy chains were used (a kind gift from Professor Dr. Jaques Neefjes, NKI, Amsterdam, The Netherlands). Normal epithelium of benign ovarian cysts and liver and renal tissue served as control. In addition, 4 *μ*m sections of the TMAs were stained with the mouse monoclonal antibodies clone MEM-E/02 (MCA2193, AbD Serotec, Kidlington, UK) and clone 4H84 (LifeSpan Biosciences, WA, USA) against HLA-E and HLA-G, respectively.

First, the tissue sections were deparaffinised and rehydrated using graded concentrations of ethanol to distilled water; endogenous peroxidise activity was blocked with 0.03% H_2_O_2_/MeOH for 20 minutes. Antigen retrieval was performed in boiling 0.01 M citrate buffer (pH 6.0) for 12 minutes. After 2 hours of cooling in citrate buffer, slides were washed twice in distilled water and twice in phosphate-buffered saline (PBS). Subsequently, incubation was performed overnight at room temperature with the primary antibodies diluted in PBS containing 1% bovine serum albumin. Second, sections were incubated with BrightVision polyhorseradish peroxidase anti-mouse/rabbit/rat IgG (Immunologic BV, Duiven, The Netherlands) for 30 minutes at room temperature. Washing between incubations was performed 3 times for 5 minutes in PBS. Immune complexes were visualized by applying a 0.05 M* tris*-HCl buffer (pH 7.6) containing 0.05% of 3,3′-diamino-benzidine-tetrahydrochloride and 0.0018% of H_2_O_2_. After 10 minutes, the reaction was stopped by rinsing with demineralised water. Finally, the tissue sections were counterstained with Mayer's haematoxylin before addition of a coverslip.

Immunohistochemical staining was scored without prior knowledge of clinical parameters and based on the intensity and percentage of positively stained tumour cells. The percentage of positively stained tumour cells was scored from 0 to 5: absent (<1%, 0), sporadic (1–5%, 1), local (6–25%, 2), occasional (26–50%, 3), majority (51–75%, 4), or large majority (>75%, 5). The staining intensity was scored from 0 to 3 to reflect negative (0), weak (1), moderate (2), or strong (3) staining intensity. Samples stained for classical HLA class I were categorized in one of three categories of expression based on the sum of the scores: normal expression, total score 7-8; weak, 3–6; and total loss, 0–2. Any variation in expression was considered as downregulation (score ≤ 6).

For HLA-G, membrane or combined membrane and cytoplasmic expression were considered positive. Scoring of HLA-G and -E was similar to classical HLA class I scoring, except that labelling of categories was different (strong expression 7-8; low expression 3–6; and no expression 0–2). Samples with any expression (>3) were considered unregulated. HLA-E positivity was also analysed using 5 or higher as cut-off as described earlier for ovarian cancer [12]. To obtain high concordance rate with whole tissue slides, a minimum of 2 cores with representative tumours tissue had to be present on the TMA to be used in statistical analyses.

### 2.4. Total RNA Isolation and Microarray Hybridization

RNA was isolated from frozen sections that contained at least 50% of tumour collected at surgery for ovarian cancer. Of these, 65 samples matched with tissue used to construct the TMAs. For this study, we used HLA-G gene expression data of these 65 matched cases.

For each specimen, 30 sections of 20 *μ*m were cut for RNA isolation. Total RNA was isolated and extracted using RNA Bee (Amsbio, UK) and the RNeasy Mini kit (DNAse-treated; Qiagen, The Netherlands) and amplified using the Illumina TotalPrep RNA Amplification kit (Ambion, Life Technologies). Samples with RIN values ≥7, as evaluated using the Bioanalyzer (Agilent, California, USA), were used for genetic analyses. RNA of the 65 samples was hybridized to Illumina Human HT-12 v4 Expression BeadChip 47 K Arrays (Illumina, CA, USA). The arrays provide intensity data for each probe or probe set, indicating a relative level of hybridization with the labelled target. Genes that were not detected above background level in at least one sample were excluded from the analysis. The data were then normalized by applying between array simple scaling (i.e., mean centring between arrays) and a subsequent log2 transformation.

### 2.5. sHLA-G Enzyme-Linked Immunosorbent Assay

Serum samples were collected from patients visiting the department of gynaecologic oncology and stored at −80°C. Samples were collected before starting the treatment, after 2 courses of chemotherapy, at the end of treatment, and at time of recurrence. Of those patients whose tissue was used to construct TMAs, we could retrieve serum of 50 patients.

sHLA-G was quantified using a commercially available ELISA kit (Exbio, Prague, Czech Republic) according to the manufacturer's instructions. Briefly, calibrators and samples were incubated in microtitration wells precoated with monoclonal antibody, mouse anti-HLA-G monoclonal antibody (MEMG/9), which recognized the most abundant soluble isoforms (shedded sHLA-G1 and intron4 containing secreted HLA-G5). After 18 hours of incubation and washing, monoclonal anti-human beta2-microglobulin antibody labelled with horseradish peroxidase (HRP), which recognized the immobilized antibody sHLA-G complex, was added to the wells and incubated for 60 minutes. Following rinsing, the substrate solution (H_2_O_2_ with tetramethylbenzidine) was added to react with the remaining HRP-conjugated antibody. After the addition of acidic stop solution, the absorbance of the resulting yellow product was measured at 450 nm using a microplate reader (BIO-RAD Model 550, CA, USA). According to absorbance values proportional to sHLA-G concentrations of calibrators, a calibration curve was constituted and used to determine the sHLA-G concentrations of serum samples. The interassay coefficient variation of the human sHLA-G ELISA kit was 1.3–16.9% and the limit of detection was 2 U/mL. The ELISA was performed in a blinded manner.

### 2.6. Statistical Analysis

Association of clinicopathologic parameters with HLA expression was analysed with two-sided Chi-square tests. To correlate loss of expression or upregulation of classical and nonclassical HLA with clinicopathologic parameters or survival, expression of classical HLA class I was analysed as normal expression versus downregulation. Nonclassical HLA was analysed as no expression versus upregulation.

Survival was expressed as progression-free survival (PFS) and disease specific survival (DSS). For DSS and PFS, the Kaplan-Meier method and log-rank test were used to calculate difference between groups with and without expression of classical and nonclassical HLA. To determine whether clinicopathologic characteristics as well as HLA class I expression were related to survival, univariable and multivariable Cox regression analyses were performed.

Correlation of sHLA-G and HLA-G expression in tissue was analysed with Spearman's rho. Differences in concentration of sHLA-G before, during, or after treatment and at recurrence were compared between matched cases using the Wilcoxon-rank test for the median. In all cases *P* values <0.05 were considered statistically significant. Statistical analysis was performed in IBM SPSS (Version 19.0, IBM SPSS, Chicago, IL).

Gene expression levels of HLA-G were analysed using R2: microarray analysis and visualization platform (http://r2.amc.nl/). Differential gene expression analysis of HLA-G of tissue with and without HLA-G protein expression and sHLA-G concentration was performed with ANOVA. For survival analyses, Kaplan-Meier method and log-rank test were used to calculate differences between groups with expression levels above and below the median.

## 3. Results

### 3.1. Patient Characteristics

Patients with high grade epithelial ovarian carcinoma treated with primary or interval debulking surgery were included (*n* = 169). From 141 patients, ovarian tissue was available of which 108 samples were collected before chemotherapy started.

Median age at diagnosis was 60 years (range 36–88 years). The majority of cases had advanced stage disease (*n* = 154) and residual disease after debulking surgery (*n* = 132). Median follow-up was 31 months (0.7–186); 125 patients had recurrence or progression. Median DSS was 38 months (95% CI 29–47) and PFS was 15 months (95% CI 12–18). Clinicopathologic characteristics are summarized in Table 1.

### 3.2. Correlation of HLA Class I Protein Expression and Clinicopathologic Characteristics

**Figure 1:**
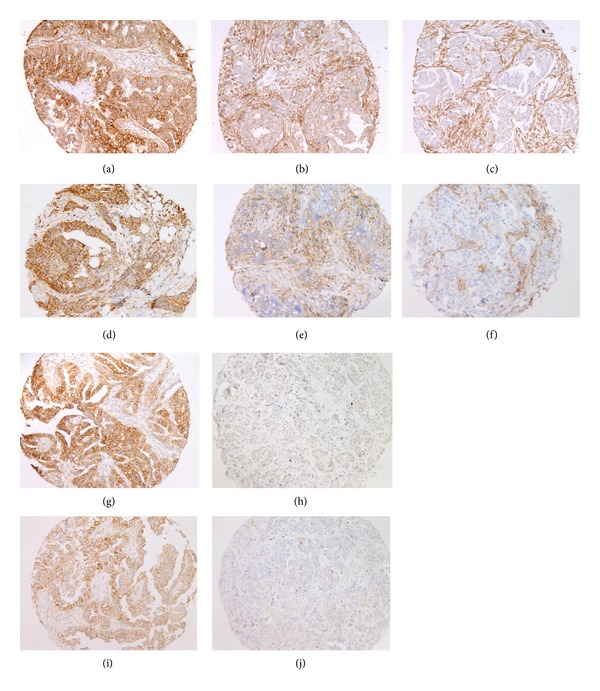
Representative examples of HLA immunohistochemical staining, HLA-A ((a)–(c)), HLA-B/C ((d)–(f)), HLA-E ((h)–(j)), and HLA-G ((i)-(j)). Strong expression ((a), (d), (g), and (i)), weak expression ((b) and (e)), and loss of expression ((c), (f), (h), and (j)).

Expression of all classical and nonclassical HLA class I molecules could be evaluated in 137 tumours (81%) (Figure 1). Downregulation of HLA-A occurred in 74.6% of the cases; in 37.2%, there was no expression at all. Downregulation of HLA-B/C was observed in 82.7%; in 46%, tumours expression was totally absent. HLA-E and HLA-G upregulation was observed in 73.4% and 47.9%, respectively. Clinical factors related to HLA protein expression were residual disease after surgery and sensitivity to chemotherapy (Table 2). In univariable analysis, residual disease after surgery (OR: 2.42; 95% CI 1.06–5.52) and sensitivity to platinum based chemotherapy (OR: 2.10; 95% CI 1.05–4.19) were significantly related to upregulation of HLA-G. In multivariable analysis sensitivity to platinum based chemotherapy (OR: 2.46; 95% CI 1.05–5.75) was independently associated with HLA-G upregulation. Furthermore, residual disease after surgery was independently associated with HLA-A downregulation (OR 2.86; 95% CI 1.17–7.03).

Besides, combined absence of HLA-A and HLA-G expression, which was correlated to residual disease after surgery (*P* = 0.038), combination of expression levels of HLA-E and -G or classical and nonclassical HLAs, did not correlate with clinicopathologic characteristics.

**Figure 2:**
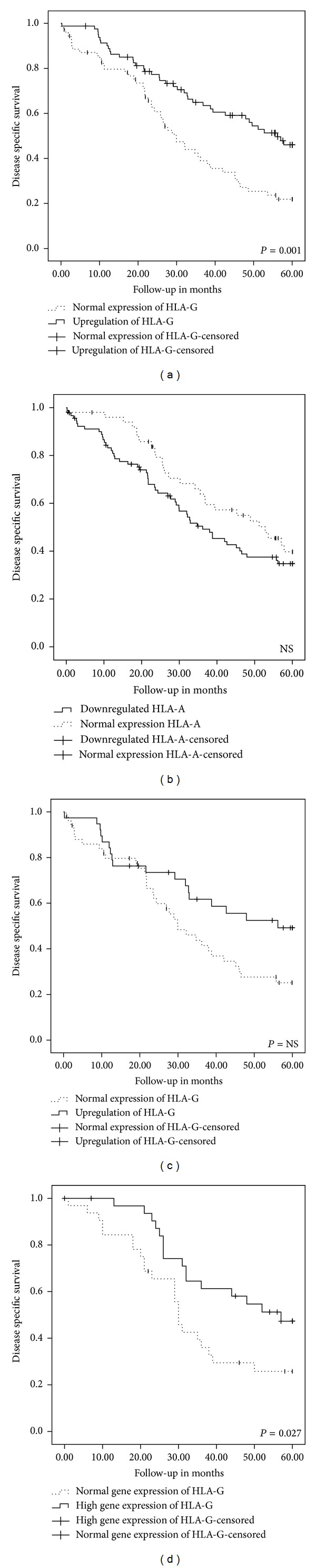
Survival analyses. Kaplan-Meier 5-year survival curves and log-rank test for HLA-G expression (a), HLA-A expression (b), HLA-G expression in tumours with downregulation of HLA-A (c), and HLA-G gene expression at the median expression cut-off of 230.4 fluorescence units (d) (NS = not significant).

When correlating HLA expression with survival, expression of HLA-G was significantly correlated with a favourable prognosis. Both PFS and DSS were significantly better when tumours expressed HLA-G. PFS was 19 months and decreased to 6 months, if there was no expression (*P* = 0.038). DSS of patients with HLA-G expression was 56 months versus 30 months, when there was no expression of HLA-G (*P* = 0.008). Five-year survival of patients with tumours expressing HLA-G was significantly better (*P* = 0.001) (Figure 2(a)). In the group of patients of whom ovarian tissue was collected before chemotherapy treatment started (*n* = 108), PFS for HLA-G positive cases was 28 months versus 16 months for those lacking expression (*P* = 0.027). DSS advantage in this group was 40 months (*P* = 0.011).

In univariate analysis, advanced stage disease (HR 4.7; 95% CI: 1.16–19.17), IDS (HR 1.8; 95% CI: 1.15–2.91), macroscopic residual tumour (HR 2.6; 95% CI: 1.57–4.43), and lack of expression of HLA-G (HR 1.69; 95% CI: 1.14–2.51) were related to worse survival. In multi-variate analysis, HLA-G expression remained an independent prognostic factor for improved survival (*P* = 0.020), as well as no residual disease after surgery and PDS. However FIGO stage did not retain prognostic significance (Table 3).

Downregulation of HLA-A resulted in a shorter PFS, 14 months (95% CI 11.1–16.9) instead of 25 months (95% CI 15.5-34.5) (*P* = 0.047), when there was normal expression. Disease specific survival at five-year follow-up was not different for patients with tumours lacking HLA-A expression (Figure 2(b)). Within the whole follow-up period, DSS was worse, when there was downregulation (36 versus 58 months); however, this was not significant (*P* = 0.066). Expression or absence of expression of HLA-B/C and -E (any upregulation or with a cut-off of 5) or combination of HLA-E/G did not influence prognosis. Also when stratified for classical HLA class I expression no difference in survival was observed according to HLA-E and/or -G expression (Figure 2(c)).

### 3.3. HLA-G Gene Expression in Frozen Biopsies and sHLA-G Concentration in Serum of High Grade EOC Patients

The median level of HLA-G gene expression in tumour tissue was 230.38 fluorescence units. Patients with high HLA-G gene expression had a significant better prognosis than those with low expression for 5-year disease specific survival (*P* = 0.027) (Figure 2(d)). Protein expression of HLA-G in tumour tissue was not correlated with HLA-G gene expression (*P* = 0.43) and also no correlation was observed between serum sHLA-G levels and protein expression (*R* = 0.26, *P* = 0.066) or sHLA-G levels and gene expression.

**Figure 3:**
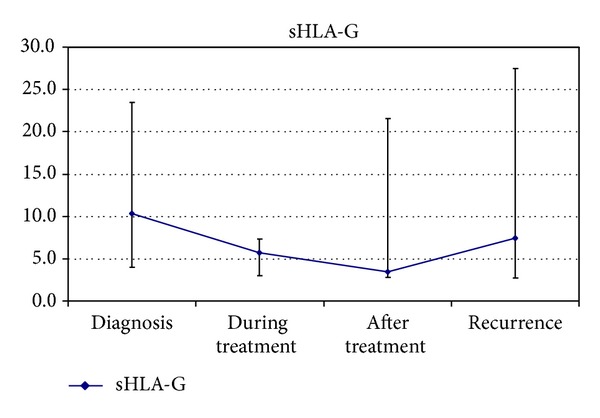
Median concentration of sHLA-G (u/mL) at diagnosis, after 2 cycles of chemotherapy, after primary treatment, and at recurrence. ⊥ is the lowest interquartile and *⊤* is the highest interquartile.

The concentration of sHLA-G in serum at start of treatment had a median of 10.3 U/mL (interquartile range (IQR) 3.0–23.5). Median concentration at the start of the treatment was higher than after 2 cycles of chemotherapy 5.7 U/mL (IQR 3.0–7.3) (*P* = 0.038). Concentration of sHLA-G decreased during the treatment and increased at recurrence (Figure 3). At recurrence, levels of sHLA-G were equally high as at the start of the treatment (median 7.4 U/mL (IQR 2.8–27.5)). Finally, no difference was seen in disease specific survival of patients with sHLA-G levels in serum above the median (*n* = 19) and below the median (*n* = 17) (*P* = 0.70).

## 4. Discussion

In this study, we analysed expression of classical and nonclassical HLA class I in high grade ovarian carcinomas. Upregulation of HLA-G expression was an independent predictor for improved survival. Furthermore, our analyses showed that HLA-G expression in these tumours is correlated with residual disease after surgery and sensitivity to platinum chemotherapy. In addition, loss of expression of HLA-A resulted in shorter progression-free survival. The expression or loss of expression of other classical and nonclassical HLA molecules in combination or alone was not associated with survival.

In concordance with protein expression, elevated gene expression of HLA-G was associated with good prognosis. However, besides an elevated concentration of sHLA-G at the start of the treatment, the concentration of serum sHLA-G did not show any prognostic relevance.

Prognostic significance of HLA class I molecules has been described in various solid as well as heamatopoetic tumours, including ovarian cancer. Several groups showed that intact HLA phenotype confers better prognosis in terms of overall survival [9, 25, 26]. Only one study, by Vitale et al. [40], did not show a survival advantage for HLA class I in ovarian cancer. Mostly, the lack of expression of classical HLA class I is associated with poor survival, whereas expression of HLA-E or -G results in a worse prognosis [12, 13, 17, 25, 35, 41, 42]. Recently, however, it was shown that not only the absence of HLA-G and -E expression but also the absence of HLA class I expression was associated with worse survival in colon cancer patients [43]. This examples the controversies which exists regarding HLA and survival, which could be due to differences in staining techniques and scoring or definition of expression.

Although expression of HLA-G is often correlated with poor survival, which is explained by inhibition of NK cell and CTL-mediated lyses, our data show that, in high grade EOC, expression of HLA-G is associated with a good prognosis. It is assumed that upregulation helps the tumour to evade the immune response of the host by blocking NK cell activity whilst others dispute this [27, 31]. Improved survival of patients with epithelial ovarian cancer expressing HLA-G in tumour cells in ascites has been described [31]. Yet, the biological significance of HLA-G remains uncertain at present.

HLA-G is more often expressed in high grade tumours [37, 44, 45]; therefore, it is thought that HLA-G plays a role in tumour growth and aggressive behaviour of high grade EOC. HLA-G is also thought to influence chemosensitivity of high grade EOC [31].

Furthermore, as described in melanoma cell lines, expression of HLA-G can account for susceptibility to NK-mediated lysis due to a switch in alternative splicing [30]. The primary transcript of HLA-G is alternatively spliced, producing seven different mRNA molecules encoding four membrane-bound (HLA-G1 to HLA-G4) and 3 soluble (HLA-G5-G7) protein isoforms. The susceptibility to NK-mediated lysis could be explained by a switch of alternative splicing leading to the loss of cell surface HLA-G1 and its replacement by intracellular HLA-G2, through which tumour cells become more susceptible to NK cell-mediated lysis [30, 35]. Recent findings show that HLA-G interacts with KIR2DL4 on NK cells to regulate the production of cytokines and chemokines [46].

This binding leads to proangiogenic factors and inflammatory cytokines production activating the immune response. To further examine how NK and T cells respond to HLA-G, we are currently analysing the expression of KIR2DL4 on CTLs and NK cells isolated from high grade EOC patients. On the contrary, recently Lin et al. described that tumour invasiveness or metastasis correlated with HLA-G expression may rely on the induction of MMP-15 expression by HLA-G in ovarian cancer [47].

Expression of nonclassical HLAs in tumours lacking expression of classical HLA has been described in several solid tumours to influence prognosis in a negative way [28, 42, 48]. This is explained by the mechanism of downregulation of classical HLA class I resulting in escape of tumours from cytotoxic T cell immune recognition. By upregulation of nonclassical HLA, they may further escape immune recognition [35]. We could not demonstrate this in the current high grade ovarian carcinoma cohort. Although we could not indisputably demonstrate a significant correlation between HLA-G gene expression and expression at the protein level, high gene expression was found to be associated with a good prognosis. There is evidence of upregulation of HLA-G mRNA in response to transformation, neovascularisation, inflammation, and infection [49]. Increased vascularity may suggest improved tumour oxygenation and drug delivery and thereby improved response to chemotherapy [50]. High gene expression of HLA-G could reflect highly vascularised tumours and susceptibility to chemotherapy.

In contrast, detection of soluble HLA-G in ascites was described to be higher in malignant ascites of ovarian and breast cancer than in ascites of benign disease [44], but detection of sHLA-G in serum of ovarian cancer patients has not been described. Although the concentration of sHLA-G at diagnosis was higher than after the treatment, in the present study, no prognostic value of sHLA-G could be demonstrated. Schütt et al. described in a subgroup of lung cancer patients with squamous cell carcinoma a better prognosis for patients with low concentrations of sHLA-G [51]. Similar to the findings in our study, no prognostic significance for sHLA-G concentration was observed in breast, renal cell, and esophageal carcinoma [52–54].

Expression of HLA-G on tumour cells in malignant effusions has been demonstrated to be related to survival and proposed as a possible marker of tumour susceptibility to chemotherapy [31]. In concordance with the findings of Davidson et al., who found reduced expression of HLA-G in effusions obtained after the start of chemotherapy, we found a reduced concentration of sHLA-G in serum in samples obtained during and after treatment. This could indicate a role in susceptibility to chemotherapy.

In order to validate the use of HLA-G expression as a predictive marker in selecting patients for treatment, independent, large, and homogeneous cohorts should be evaluated to make definite conclusions on the prognostic influence of HLA expression in EOC.

In conclusion, this is the first study to describe the correlation of classical and nonclassical HLA class I expression levels with survival in high grade epithelial ovarian cancer and to correlate both membrane-bound and soluble HLA-G protein and HLA-G gene expression. The here presented data show that, in high grade epithelial ovarian carcinomas, HLA-G expression is an independent parameter for improved survival.

## Figures and Tables

**Table 1:** Patient characteristics.

Baseline characteristics	All patients (*n* = 169)
Mean age, years (SD)	61 (11.7)
FIGO stage	
I	8 (5%)
II	7 (4%)
III	125 (74%)
IV	29 (17%)
Histologic classification	
High grade serous	141 (83%)
Undifferentiated	28 (17%)
Residual disease after debulking surgery	
No macroscopic tumour	37 (22%)
Less than 1 cm residual disease	46 (27%)
1 cm or more residual disease	86 (51%)
Kind of debulking surgery	
PDS	134 (79%)
IDS	35 (21%)
Tissue origin	
Ovary	141
Metastases	28
Kind of chemotherapy given	
None	9 (5%)
Single drug platinum	5 (3%)
Multidrug carboplatin/paclitaxel	123 (19%)
Multidrug with platinum	32 (73%)
Amount of cycles	6 (5–7)
Platinum sensitivity	
Refractory disease	34 (20%)
Platinum resistant disease	36 (21%)
Partial chemosensitive	25 (15%)
Platinum sensitive disease	65 (38%)

Refractory disease: recurrence occurred during chemotherapy treatment; platinum resistant disease: recurrence occurred within 6 months after the last cycle of chemotherapy; partial platinum sensitive disease: recurrence occurred after 6 months or within 1 year after last cycle of platinum based chemotherapy; platinum sensitive disease: recurrence occurred more than 1 year after the last cycle of chemotherapy.

**Table 2:** Correlation of HLA-A, HLA-B/C, HLA-G, and HLA-E expression with clinical parameters.

	HLA-A			HLA-B/C			HLA-E			HLA-G		
	Normal expression	Downregulation	*P*	Normal expression	Downregulation	*P*	Upregulation	Normal expression	*P*	Upregulation	Normal expression	*P*
*N* (%)	51 (36)	91 (64)		29 (17)	124 (83)		124 (83)	28 (17)		81 (53)	71 (47)	
Variables												
Age												
<60	30	42	0.15	14	63	0.78	65	10	0.26	40	38	0.61
>60	21	49		12	61		59	15		33	41	
FIGO stage												
Stages I-IIA	4	4	0.39	1	6	0.83	5	2	0.39	3	3	0.87
Stage >IIB	47	87		25	118		119	23		78	68	
Residual tumor												
No residual	18	14	**0.006**	11	22	**0.006**	27	4	0.51	23	10	**0.033**
Any residual	33	77		15	102		97	21		58	61	
Platinum sensitivity												
Resistant	15	40	0.08	9	48	0.66	52	9	0.66	25	35	**0.014**
Sensitive	33	46		15	65		66	14		53	32	

**Table 3:** Cox regression analysis on disease specific survival including clinicopathological factors and HLA expression.

Disease specific survival	Univariable analysis			Multivariable analysis		
	HR	95% CI	*P* value	HR	95% CI	*P* value
Age (years)	1.01	0.99–1.03	0.185			
FIGO stage						
Low (I-IIA)	1			1		
High (IIB–IV)	4.70	1.16–19.17	0.030	3.74	0.48–28.99	0.207
Kind of surgery						
PDS	1			1		
IDS	1.80	1.15–2.91	0.011	2.48	1.49–4.15	0.001
Residual disease						
Microscopic	1			1		
Macroscopic	2.60	1.57–4.43	<0.000	2.21	1.21–4.02	0.009
HLA-A expression						
Normal	1					
Downregulation	1.30	0.85–1.98	0.231			
HLA-B/C expression						
Normal	1					
Downregulation	1.13	0.68–1.88	0.647			
HLA-E expression						
Normal	1					
Upregulation	1.31	0.75–2.27	0.341			
HLA-G Expression						
Normal	1.69	1.14–2.51	0.009	1.62	1.08–2.42	0.020
Upregulation	1					

HR: hazards ratio, CI: confidence interval, Ref.: referent, PDS: primary debulking surgery, IDS: interval debulking surgery.
